# Utilization of carbon catabolite repression for efficiently biotransformation of anthraquinone O-glucuronides by *Streptomyces coeruleorubidus* DM

**DOI:** 10.3389/fmicb.2024.1393073

**Published:** 2024-04-16

**Authors:** Chen Tao, Quyi Wang, Junyang Ji, Ziyue Zhou, Bingjie Yue, Ran Zhang, Shu Jiang, Tianjie Yuan

**Affiliations:** ^1^School of Pharmacy, Nanjing University of Chinese Medicine, Nanjing, China; ^2^Jiangsu Collaborative Innovation Center of Chinese Medical Resources Industrialization, Nanjing University of Chinese Medicine, Nanjing, China

**Keywords:** anthraquinone, glucuronidation, *Streptomyces*, biotransformation, carbon catabolite repression

## Abstract

Carbon catabolite repression (CCR) is a highly conserved mechanism that regulates carbon source utilization in *Streptomyces*. CCR has a negative impact on secondary metabolite fermentation, both in industrial and research settings. In this study, CCR was observed in the daunorubicin (DNR)-producing strain *Streptomyces coeruleorubidus* DM, which was cultivated in high concentration of carbohydrates. Unexpectedly, DM exhibited a high ability for anthraquinone glucuronidation biotransformation under CCR conditions with a maximum bioconversion rate of 95% achieved at pH 6, 30°C for 24 h. The co-utilization of glucose and sucrose resulted in the highest biotransformation rate compared to other carbon source combinations. Three novel anthraquinone glucuronides were obtained, with purpurin-O-glucuronide showing significantly improved water solubility, antioxidant activity, and antibacterial bioactivity. Comparative transcript analysis revealed that glucose and sucrose utilization were significantly upregulated as DM cultivated under CCR condition, which strongly enhance the biosynthetic pathway of the precursors Uridine diphosphate glucuronic acid (UDPGA). Meanwhile, the carbon metabolic flux has significantly enhanced the fatty acid biosynthesis, the exhaust of acetyl coenzyme A may lead to the complete repression of the biosynthesis of DNR, Additionally, the efflux transporter genes were simultaneously downregulated, which may contribute to the anthraquinones intracellular glucuronidation. Overall, our findings demonstrate that utilizing CCR can be a valuable strategy for enhancing the biotransformation efficiency of anthraquinone O-glucuronides by DM. This approach has the potential to improve the bioavailability and therapeutic potential of these compounds, opening up new possibilities for their pharmaceutical applications.

## Introduction

*Streptomyces* are renowned for their remarkable ability to produce a wide array of secondary metabolites with diverse chemical structures and bioactivities (Sun et al., 2020). The biosynthesis of these secondary metabolites is traditionally tightly controlled by environmental cues and nutrient availability (van der Heul et al., 2018).

Carbon catabolite repression (CCR) is a highly conserved mechanism that allows bacteria, including *Streptomyces*, to prioritize the utilization of preferred carbon sources over others (Gorke and Stulke, 2008; Nair and Sarma, 2021). However, it has been observed that high concentrations of carbon sources can significantly interfere with the formation of secondary metabolites. Several studies have reported the suppression of over 30 different secondary metabolites in *Streptomyces* when exposed to various carbon sources such as glucose, maltose, and sucrose (Romero-Rodriguez et al., 2017). As a result, CCR is generally considered an adverse condition for industrial strains (Ruiz-Villafan et al., 2021). Numerous efforts have been made to alleviate CCR and enhance industrial performance. For instance, the production of natamycin, an antifungal compound, was increased by 1.6-fold through the implementation of slow glucose feeding, which helped alleviate the effects of CCR (Elsayed et al., 2019). Additionally, the deletion of an α-tubulin gene (tubB) involved in CCR regulation in the fungus *Trichoderma reesei* promoted the production of cellulase and hemicellulase enzymes (Shibata et al., 2021). Although CCR is generally associated with the repression of microbial fermentation production, it also induces significant changes in carbon metabolic flux and regulatory systems, which may have positive effects. However, the utilization of CCR remains largely unexplored.

Anthraquinones with cyclic diketone structures, specifically 9,10-dioxoanthracenes, are a prominent group of biologically active compounds derived from traditional Chinese medicine (Li and Jiang, 2018). These anthraquinones have gained significant interest due to their remarkable bioactivities, which encompass anti-cancer, anti-bacterial, anti-inflammatory, and antioxidant properties (Malik and Muller, 2016). Nevertheless, their limited water solubility and low oral bioavailability have impeded their wider utilization and application. Glycosylation is the most commonly observed modification in natural products (Shrestha et al., 2019). Glucuronidation is widely distributed in plants, where glucuronic acid is conjugated to plant-derived aglycones such as baicalein-7-O-β-glucuronide, which exhibits health-promoting activities (Docampo et al., 2017). Scutellarin-7-O-glucuronide, the primary component of the Chinese medicine breviscapine, has been extensively used in clinical treatment for cardiovascular diseases (Wang et al., 2013). Glucuronidation is typically a phase II metabolic process, considered a detoxification step in the mammalian system (Stachulski and Meng, 2013). However, glucuronide metabolites often possess enhanced water solubility and polarity compared to their corresponding aglycones, indicating their potent pharmacological activities (Docampo-Palacios et al., 2020). For instance, morphine-6-O-glucuronide demonstrates a 100-fold stronger anesthetic effect than morphine, as evidenced by clinical trials (Kilpatrick and Smith, 2005). Moreover, glucuronides can be developed as water-soluble prodrugs to overcome the limitations of cytotoxicity and low solubility in the parent compounds (Jarlstad Olesen et al., 2020). Chemical synthesis of glucuronides often involves several challenging steps, including stereo-selectivity protection and deprotection of functional groups (Walther et al., 2019). On the other hand, the practical application of purified enzymes as biocatalysts *in vitro* presents challenges. Purified enzymes are typically unstable and prone to denaturation, and the sugar donor UDPGA (uridine diphosphate glucuronic acid) is expensive (2000 USD/g) (Ohnuki et al., 2019). Therefore, whole-cell biocatalysis for glucuronidation represents a green and cost-effective method for generating valuable bioactive compounds.

*Streptomyces coeruleorubidus* DM is an industrial strain commonly employed for the production of the anthracycline anticancer drug Daunorubicin (DNR) (Yuan et al., 2011). Previous studies have primarily focused on elucidating the biosynthetic pathway of DNR. However, in the present study, it was unexpectedly discovered that DM possesses the ability to glucuronidate anthraquinones in synthetic F media containing 10 g/L glucose and 20 g/L sucrose, which can be attributed to carbon catabolite repression (CCR) effect. The biotransformation conditions were established and optimized, and various bioconversion substrates were explored. Novel anthraquinone glucuronides were purified, identified, and further investigated for their bioactivities. Additionally, the anthraquinone glucuronidation mechanism of DM under CCR conditions was analyzed through comparative transcription sequencing. This study highlights the potential application of CCR for the bioconversion of valuable products.

## Materials and methods

### General procedures

Anthraquinones (emodin, aloe-emodin, anthraflavic acid, purpurin, alizarin, 1,8-dihydroxyanthraquinone, 1,4-diaminoanthraquinone, and 2-aminoanthraquinone) and a Hydroxyl Free Radical Scavenging Capacity Assay Kit were purchased from Sangon Biotech Co., Ltd. (Shanghai, China). All other chemicals and reagents were the highest chemical grade. MS solid media was used for culture of DM colonies and spores. Trypticase Soy Broth (TSB) media was used for seed growth of DM. Four different culture media were used for fermentation and biotransformation of DM (Wang et al., 2018): A culture media (starch 10 g/L, yeast extract 4 g/L, peptone 2 g/L, CaCO_3_ 1 g/L, Fe_2_(SO_4_)_3_ 4H_2_O 40 mg/L, KBr 100 mg/L), P culture Media (yeast extract 2 g/L, mannitol 4 g/L, peptone 2 g/L), F culture media (sucrose 20 g/L, glucose 10 g/L, casamino acid 0.1 g/L, yeast extract 5 g/L, Mops 5 g/L, trace elements 1 mL, K_2_SO_4_ 0.25 g/L, MgCl_2_ 6H_2_O 1 g/L), and Y culture media (yeast extract 4 g/L, malt extract 10 g/L, glucose 4 g/L). *Bacillus subtilis* and *Escherichia coli* (laboratory preservation) were cultured in LB at 37°C overnight for the basic antibacterial assay.

### Culture preparation and whole-cell biotransformation

Fresh spores of DM was inoculated into 200 mL TSB and incubated at 30°C, 160 rpm for 2 days. The fermentation process was as follows: approximately 20 mL seed culture was transferred to 200 mL fermentation media and incubated at 30°C, 160 rpm for 5 days. The biotransformation process was as follows: approximately 20 mL seed culture was transferred to 200 mL fermentation media F fermentation media and incubated at 30°C, 160 rpm for 48 h. Anthraquinone were dissolved in dimethyl sulfoxide at the concentration of 30 mg/mL for exogenous supply to DM culture. Each substrate was applied to a 48 h culture of DM and transformed into the respective products by incubating for 48 h. Subsequently, all cultures were harvested and centrifuged at 8,000 rpm for 10 min. The supernatants were mixed with an equal volume of ethyl acetate and extracted twice. The organic layers were evaporated by rotary evaporator and dissolved in 1 mL methanol. The resulting samples were analyzed by high performance liquid chromatography (HPLC) and high resolution quadruple time-of-flight electrospray ionization-mass spectrometry (HR-QTOF/MS).

### Analytical procedures

A 20 μL volume of the prepared samples was injected and analyzed by HPLC (Agilent 1260 Infinity II) using a reverse phase C18 column. The binary mobile phase was composed of solvent A (water adding 0.1% formic acid) and solvent B (100% methanol). The flow rate was 0.8 mL/min for a 23 min gradient elution program. The methanol concentrations were 10% (0 min), 10–100% (0–13 min), 100% (13–18 min), and 10% (18–23 min). Transformation products were separated and purified by Pre-HPLC with a C18 column linked to a UV detector analyzed at 280 nm using a 23 min gradient elution program with flow rate kept as 2 mL/min. The gradient elution program comprised methanol concentrations of 40% (0 min), 40–90% (0–15 min), 90% (15–19 min), and 10% (19–23 min). The purified metabolites were dissolved in dimethyl-sulfoxide-*d*6 for structural elucidation. ^1^H and ^13^C was analyzed by Nuclear magnetic resonance (NMR) by using a 500 MHz Bruker BioSpin NMR instrument. Structures of the novel compounds were elucidated by using the MestReNova 11.0 program.

### Relative water solubility assay

The water solubility of anthraquinones were analyzed by the saturation aqueous solution method. Dissolved the extra quantity anthraquinones in distilled water and agitated at room temperature for 24 h. The soluble parts were centrifuged and further analyzed by HPLC (Wang et al., 2020).

### Hydroxyl free radical scavenging capacity assay

The degree of inhibition reflects the ability of the sample to scavenge hydroxyl radicals. Briefly, 150 μL anthraquinones samples (1 mg/mL in DMSO) was added to the reaction system and kept at 37°C for 60 min. Absorbance values were then determined at 536 nm. The ability to scavenge hydroxyl radicals (D%) was calculated as: D% = (A_sample_ − A_control_)/(A_empty_ − A_control_).

### Bacterial inhibition rate assay

*E. coli* and *B. subtilis* were cultured in LB broth overnight at 37°C and 160 rpm, then were diluted to 10^3^ CFU/mL. Briefly, 50 μL anthraquinones samples (2 μM in DMSO) were added into 96-well plates and 50 μL diluted bacterial culture was added per well. The plates were kept at 37°C for 24 h, then absorbance values were detected at 600 nm. Penicillin was used as the positive control, sterile water was the negative control, and the wells only with LB media was the blank control. The calculation formula of Inhibition Rate as follows:


$$\begin{array}{l} IR\;\left(\%\right)=\left(OD600_{\mathrm{negtive}}-OD600_{\mathrm{sample}}\right)\\ \;/\left(OD600_{\mathrm{negative}}-OD600_{\mathrm{blank}}\right) \end{array}$$

### RNA extraction and sequencing

At the end of the cultivation, DM cells from two synthetic media (F and Y) were harvested by centrifugation at 10,000 rpm, 4°C for 10 min in RNase free tubes, the precipitate was instantly frozen in liquid nitrogen and deposited at −80°C before RNA extraction (Tan et al., 2020). Transcriptome experiments were carried out in three parallel samples. Transcriptome sequencing was undertaken on an Illumina HiSeq 2000 Sequencer (Illumina, United States) was subsequently performed by Majorbio Bioinformatics Technology Co., Ltd. (Shanghai, China), and the data were further analyzed on the online platform of Majorbio Cloud Platform.^1^ The resulting high-quality clean reads were mapped with the *S. coeruleorubidus* reference sequence (GenBank accession number GCA_008705135.1) by Bowtie2 software to get position and characteristic information. RNA sequencing data were further analyzed and transcript expression levels were determined by computing (fragments per kilobase of transcript per million mapped reads) FPKM.

### Statistical analysis

Measured data were stated as the mean ± standard deviation. Statistical analysis by T-tests was employed by GraphPad software. A confidence level of 95% was chosen to determine the significance, and *p* < 0.05 represents the significant statistical difference.

## Results

### The fermentation characteristics of DM cultivated with different media

**Figure 1:**
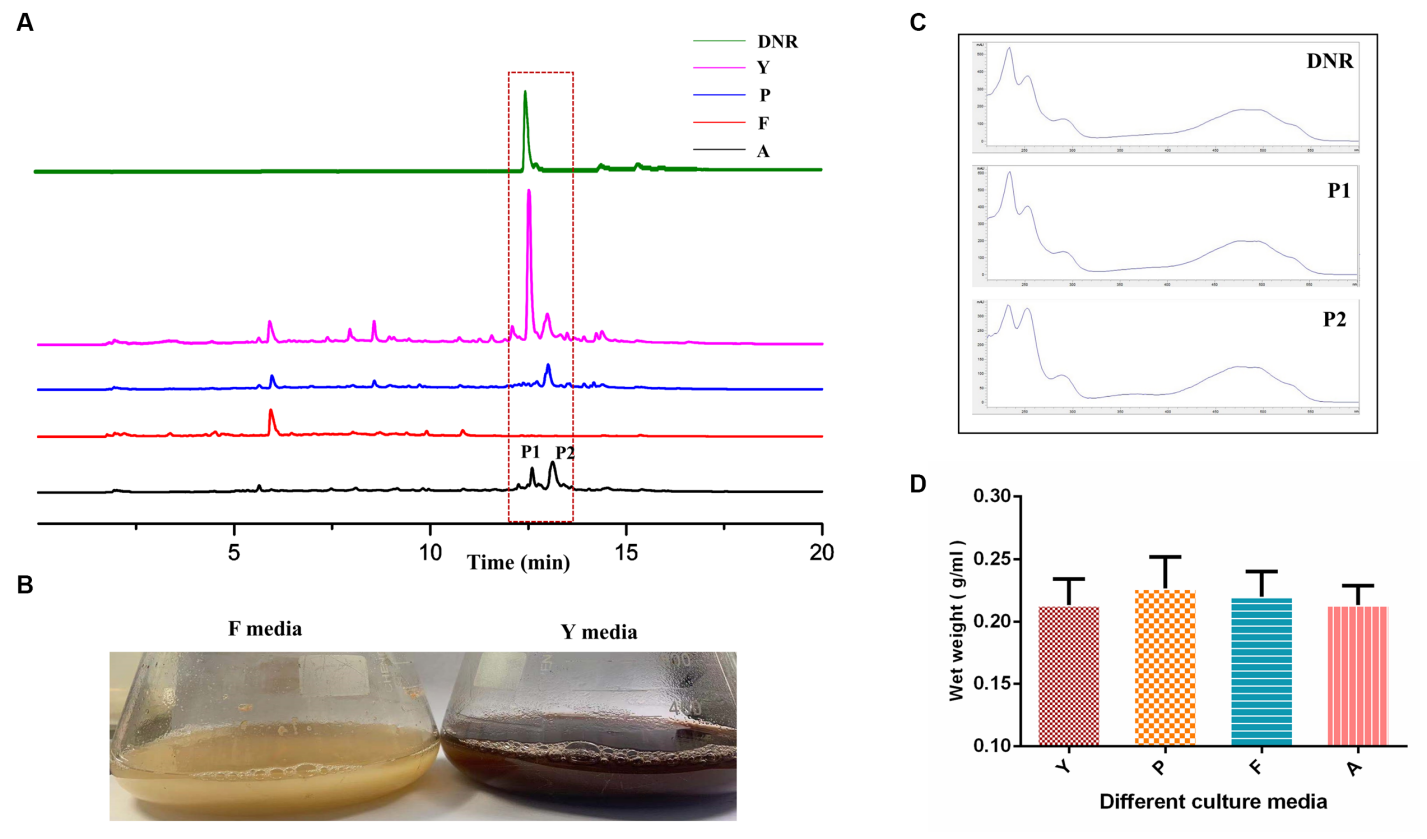
The fermentation characteristic of DM cultivated with different media. **(A)** The fermentation performance of DM cultivated in Y, P, F, A media. **(B)** The difference of culture broth in F media and Y media. **(C)** The UV scanning of the fermentation products of peak1 and peak2 in different media. **(D)** The determination of DM biomass at the end of fermentation in different media.

Four commonly used *Streptomyces* culture media, namely A, F, P, and Y, were selected for the fermentation of DM. The production of DNR, an anthracycline anticancer drug, was observed in fermentation media Y, A, and P, with the highest production observed in Y media (Figure 1A). The DM culture broth of F media showed faint yellow which was different from the other three culture media (Figure 1B). And no DNR (peak P1) and DNR-related compound (peak P2) were detected in F media (Figure 1C). However, there were no significant differences observed in the final biomass concentrations among the different culture media (Figure 1D), indicating that the variation in media did not affect the growth of DM cells.

### Establishing whole-cell biocatalysis for anthraquinone glucuronides

**Figure 2:**
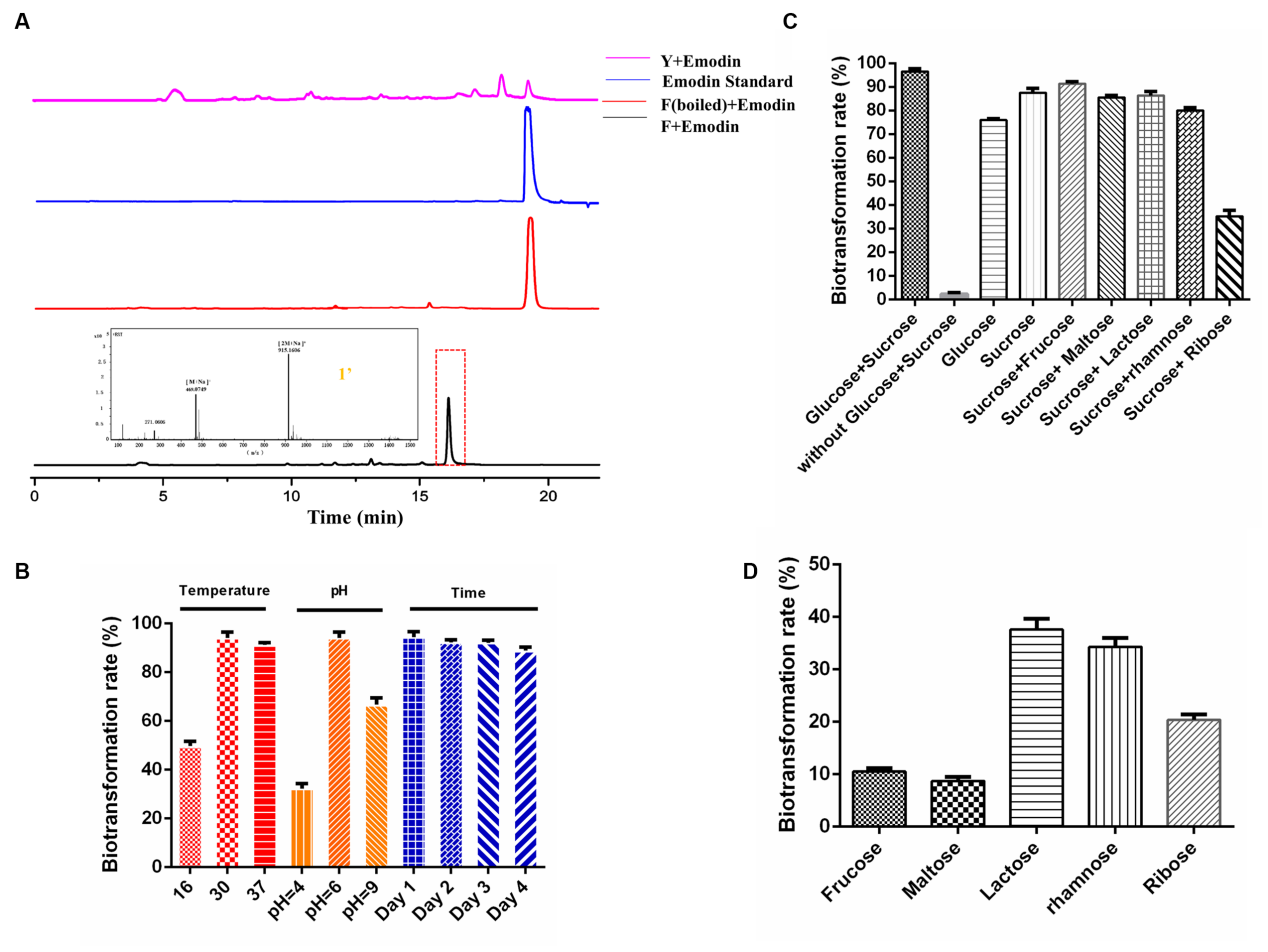
Establishing whole-cell biocatalysis of anthraquinone glucuronides under CCR condition. **(A)** HPLC results of emodin biotransformation in Y media, F media (boiled), F media, and mass data of anthraquinone glucuronide 1′. **(B)** Effects of the temperature, pH, biotransformation time on bioconversion rates. **(C)** Effects of sugar combination on biotransformation rates. **(D)** Effects of single carbon source on biotransformation rates.

The primary fermentation product of DM is anthracyclines, which have a basic anthraquinone structure. To explore the biotransformation potential, the representative anthraquinone compound emodin was exogenously supplemented to the DM broth. After two days of the biotransformation process, DM generated a new product peak, indicating the occurrence of biotransformation in F media (Figure 2A). No biotransformation products were observed when DM was cultured in Y media or when the broth was boiled before emodin supplementation, confirming that the new product was specifically derived from the biotransformation process in F media. The exact mass of the corresponding peak was [M + Na]^+^ = 469.0746, and the molecular formula was calculated as C_21_H_18_O_11_, suggesting the possible presence of emodin-like glucuronide in the product.

Furthermore, the influence of culture conditions such as temperature, pH and incubation time on emodin transformation were investigated. The biotransformation rate of anthraquinones was significantly influenced by temperature, with a peak rate of approximately 93% observed at 30°C. DM also exhibited the highest biotransformation rate at the natural pH of F media (pH = 6). The rate reached its maximum at 24 h, and the substrate was completely utilized within this time frame (Figure 2B). The effect of different carbon sources on biotransformation was also investigated. The biotransformation ability of DM was completely abolished in F media lacking glucose and sucrose. When the media was supplemented with either glucose or sucrose alone, DM still exhibited biotransformation ability, albeit with reduced bioconversion rates of 75.9 and 87.4%, respectively (Figure 2C). The combination of sucrose with five other sugars (the final concentration is 10 g/L) was also tested, and the results showed a decline in transformation rate compared to the combination of glucose and sucrose. Furthermore, when F media was supplemented with only fructose, maltose, lactose, rhamnose or ribose (the final concentration is 10 g/L), the glucuronidation bioconversion rate significantly decreased (Figure 2D).

### Exploration of the substrate selectivity for novel anthraquinone glucuronides

**Figure 3:**
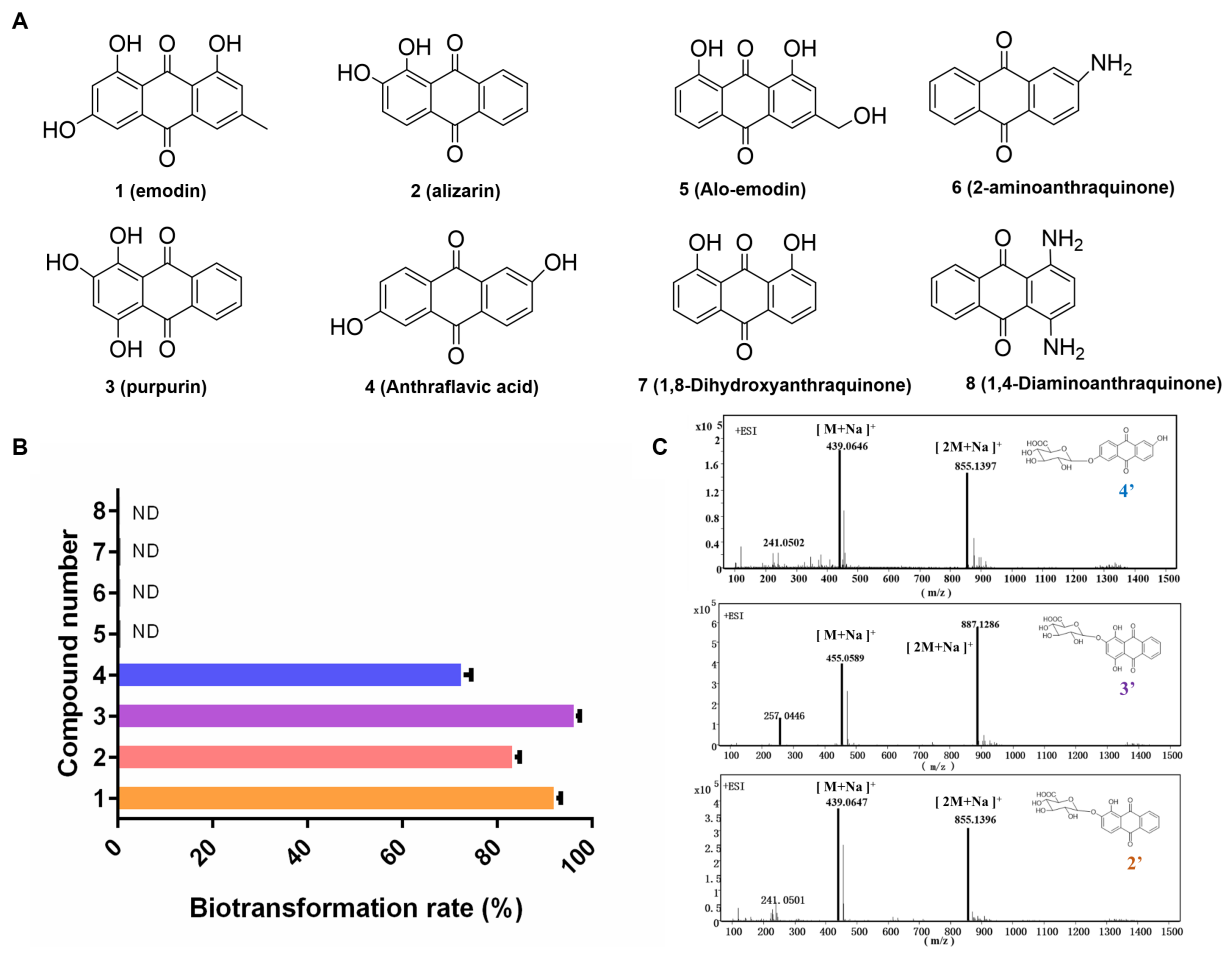
Exploration the substrate selectivity for novel anthraquinone glucuronides. **(A)** Eight substrates from Chinese medicine and synthetic compounds. **(B)** The biotransformation rates of compounds 1–8. **(C)** The mass data of three novel anthraquinone glucuronides 2′,3′,4′.

In this study, the biotransformation potential of three additional common anthraquinones derived from natural products including alizarin (2), purpurin (3), and alo-emodin (5), as well as four synthetic anthraquinone compounds including anthraflavic acid (4), 2-aminoanthraquinone (6) 1,8-dihydroxyanthraquinone (7), and 1,4-diaminoanthraquinone (8) in Figure 3A were further investigated under the optimal catalytic conditions previously established. Based on HPLC-HRMS analysis, four substrates were successfully converted into their corresponding glucuronides by DM. The highest conversion rate was observed for purpurin, with almost 96.5% of purpurin specifically transformed into its glucuronide compounds (Figures 3B,C). However, DM showed no catalytic activity towards compounds 5 to 8.

### Preparation and structural elucidation of anthraquinone O-glucuronides

To obtain the novel anthraquinone glucuronides, a 2 L DM whole-cell biotransformation was conducted for each convertible anthraquinone. All the chemical structure of the four glucuronidation product by transformation were elucidated and the product purpurin glucuronide was chosen for structure identification as the example. The anomeric proton H-1″ appeared at 5.39 ppm with a *J* value of 10 Hz. Additionally, the H-2-5″ signals were detected at 3.37–4.09 ppm. The anomeric carbon C-1″ signal was observed at 99.3 ppm, while the C-6″ signal was at 170.6 ppm. These findings indicated the presence of a glucuronic acid moiety with a β-configuration. Based on MS and 1H, 13C NMR analyses, purpurin-2-O-glucuronide (2′), alizarin-2-O-glucuronide (3′), and anthraflavic acid-2-O-glucuronide (4′) were identified as novel compounds. Emodin-3-O-glucuronide (1′) was identified as a known compound (Supplementary Figures S1–S4), which is consistent with previous literature (Yue et al., 2019).

### Pharmacological activity assay of biotransformed anthraquinone glucuronides

**Figure 4:**
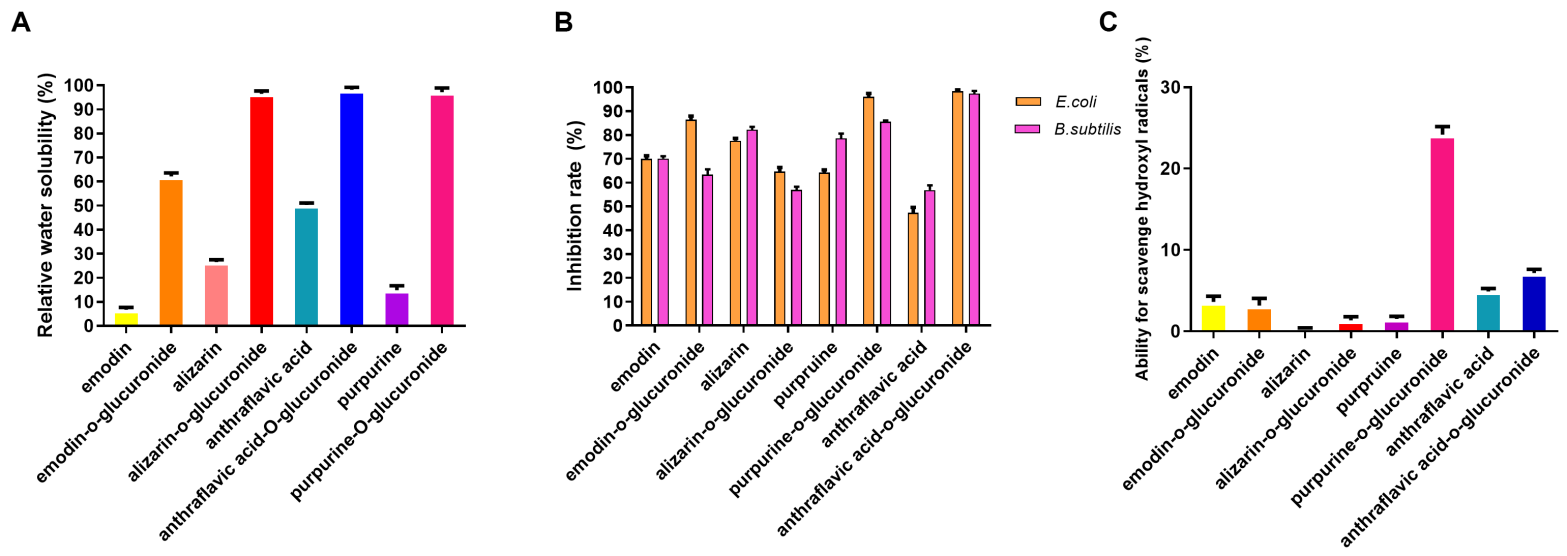
Pharmacological activity assay of biotransformed anthraquinone glucuronides. **(A)** Relative water solubility of the anthraquinone and glucuronidated products. **(B)** Antibacterial efficiency of anthraquinone and glucuronidated products. **(C)** Ability for hydroxyl radical scavenging of anthraquinone and glucuronidated products.

Compared to the anthraquinone aglycones, the water solubility of the anthraquinone glucuronidated products exhibited a significant increase. Specifically, the of water solubility of emodin-O-glucuronide and purpurin-O-glucuronide increased nearly sevenfold, from 8.5 to 60% for emodin-O-glucuronide and from 14 to 96% for purpurin-O-glucuronide (Figure 4A). The pharmacological activities of the glucuronidated products were evaluated in an antibacterial assay using *E. coli* and *B. subtilis* (Figure 4B). At a concentration of 2 μM, purpurin-O-glucuronide exhibited an inhibition rate of 84.8% against *E. coli* and 94% against *B. subtilis*. Additionally, the inhibition rate of anthraflavic acid-O-glucuronide on *E. coli* and *B. subtilis* was significantly increased by 64.4 and 97.7%, respectively, compared to anthraflavic acid. The ability of the anthraquinone glucuronides to scavenge hydroxyl radicals was also investigated (Figure 4C). Notably, purpurin-O-glucuronide exhibited a 17-fold increase in hydroxyl radical scavenging compared to the corresponding aglycone. Overall, our findings suggest that anthraquinone glucuronidation has the potential to enhance the bioactivity of the respective aglycones.

### The mechanism of anthraquinone glucuronidation driven by CCR

To investigate the mechanism underlying DM’s response to different media, we analyzed the transcriptome sequences of DM cultivated in Y media (DM-Y) and F media (DM-F). The aim was to shed light on the active metabolic pathways under carbon catabolite repression (CCR) conditions and understand why F media facilitated anthraquinone glucuronidation. A total of 3,502 genes exhibited differential expression between the two groups, with 1823 genes being upregulated when DM was cultivated in F media.

### The pathway of carbohydrate metabolism

The significant changes in transcript expression involved in carbohydrate metabolism under different culture media are summarized in Table 1. In *Streptomyces*, glucose is transported by the sugar permease GlcP, a member of the major facilitator superfamily (MFS), or directly phosphorylated by glucose kinase (GlkA) to form glucose 6-phosphate (van Wezel et al., 2005; Romero-Rodriguez et al., 2016a). Comparative transcript sequences revealed a marked increase in the genes encoding GlcP and GlkA (log_2_FC 2.16- and 2.42-fold, respectively). The maltose/maltodextrin ATP-binding cassette (ABC) transporter has been described as implicated in sucrose uptake (Zeng and Burne, 2013), and the internalized sucrose is further metabolized by sucrose-6-phosphate hydrolase, which converts sucrose into glucose-6-phosphate and fructose (Williams et al., 2019). The gene encoding sucrose-6-phosphate hydrolase in DM was significantly upregulated by 1.63-fold in F media. The hydrolyzed glucose-6-phosphate and free fructose from sucrose could provide sufficient precursors for glycolysis. Notably, the genes encoding phosphoenolpyruvate carboxykinase and fructose-1,6-bisphosphatase, which catalyze the rate-controlling step of gluconeogenesis, were significantly repressed (−4.05- and −1.81-fold, respectively) in the DM-F group. This suggests that there is no need for additional glucose synthesis to provide energy for bacterial growth when carbon sources are abundant. Additionally, the expression of two crucial rate-limiting enzymes of the pentose phosphate pathway (PPP) was significantly increased in the DM-F group (Table 1). These enzymes include glucose-6-phosphate dehydrogenase (1.05-fold increase) and 6-phosphogluconate dehydrogenase (2.37-fold increase).

**Table 1:** Changes in transcript expression of genes related to glycolysis and gluconeogenesis.

Pathway	Definition	ID	Log_2_Fold change	Adjusted-*p*-value
Glucose transport	GlkA	AJHOBMPL_01112	2.42	5.59 × 10^−28^
	GlcP	AJHOBMPL_06679	2.16	0.000872
Sucrose transport	Carbohydrate ABC transporter permease	AJHOBMPL_00151	1.22	0.1432
	Sucrose-6-phosphate hydrolase	AJHOBMPL_00758	1.63	0.007832
Glycolysis	Glucose-6-phosphate isomerase	AJHOBMPL_07716	2.68	3.27 × 10^−21^
	ATP-dependent 6-phosphofructokinase	AJHOBMPL_07506	3.51	2.14 × 10^−10^
	Glyceraldehyde-3-phosphate dehydrogenase	AJHOBMPL_03212	3.88	5.77 × 10^−97^
	Phosphoglycerate kinase	AJHOBMPL_02295	1.30	3.72 × 10^−11^
	Phosphoglycerate mutase	AJHOBMPL_08608	2.06	0.003382
	Enolase	AJHOBMPL_03786	1.18	1.93 × 10^−11^
Pentose phosphate pathway	Glucose-6-phosphate dehydrogenase	AJHOBMPL_02278	1.05	2.64 × 10^−16^
	6-phosphogluconate dehydrogenase	AJHOBMPL_04652	2.37	1.98 × 10^−42^
	Ribose-5-phosphate isomerase	AJHOBMPL_03156	1.82	5.27 × 10^−20^
UDPGA synthetic pathway	Phosphoglucomutase	AJHOBMPL_05867	2.20	2.3 × 10^−32^
	UTP-glucose-1-phosphate uridylyltransferase	AJHOBMPL_01483	1.22	2.71 × 10^−11^
	UDP-glucose-6-dehydrogenase	AJHOBMPL_07948	0.83	0.034418
	UDP-glucoronosyl transferase	AJHOBMPL_07177	1.09	7.58 × 10^−11^
Gluconeogenesis	Phosphoenolpyruvate carboxykinase	AJHOBMPL_05917	−4.05	1.2 × 10^−12^
	Fructose-1,6-bisphosphatase	AJHOBMPL_06005	−2.36	3.33 × 10^−45^

Furthermore, the gene AJHOBMPL_05867 encoding phosphoglucomutase and AJHOBMPL_01483 encoding UTP-glucose-1-phosphate uridylyltransferase were significantly upregulated in the DM-F group (2.2- and 1.2-fold, respectively). These enzymes catalyze the conversion of glucose-6-phosphate into UDP-1-glucose, which is further converted into UDP-glucuronic acid by UDP-glucose 6-dehydrogenase (0.8-fold upregulated in DM-F group). UDP-glucuronic acid can be used for the synthesis of crucial precursors for anthraquinone glucuronidation (Bar-Peled et al., 2004). Moreover, UDP-glucuronosyltransferase (UGT) increased by 1.09-fold, which catalyzes the glucuronidation of the aglycone, resulting in the formation of a β-D-glucuronide product. The upregulation of glucuronidation biosynthetic pathway in F media (Table 1), which may provide the sufficient precursor UDPGA for glucuronidation by DM.

### The pathway of TCA cycle and oxidative phosphorylation

The tricarboxylic acid (TCA) cycle is a fundamental metabolic pathway for energy generation. In the DM-F group, most of the genes involved in the TCA cycle exhibited notable upregulation, as shown in Table 2. These include pyruvate dehydrogenase (3.30-fold increase), citrate synthase (1.28-fold increase), aconitate hydratase (2.46-fold increase), and succinate dehydrogenase (2.4-fold increase). Oxidative phosphorylation, which involves electron transfer chains, is an important process for energy generation. However, the genes involved in oxidative phosphorylation and electron transport were significantly downregulated in the DM-F group. These include NADH-quinone oxidoreductase, cytochrome bc1, cytochrome c oxidase, and ATP synthase. This suggests that the process of oxidative phosphorylation was blocked when DM was cultivated in high concentrations of glucose and sucrose.

**Table 2:** Changes in transcript expression of genes related to TCA cycle and oxidative phosphorylation.

Pathway	Definition	ID	Log_2_Fold change	Adjusted-*p*-value
TCA cycle	Citrate synthase	AJHOBMPL_06902	1.28	5.81 × 10^−9^
	Aconitate hydratase	AJHOBMPL_07027	2.46	1.51 × 10^−16^
	Succinate dehydrogenase	AJHOBMPL_05805	2.40	2.11 × 10^−24^
	Pyruvate dehydrogenase	AJHOBMPL_04708	3.30	1.77 × 10^−83^
Oxidative phosphorylation	NADH-quinone oxidoreductase	AJHOBMPL_05489	−4.71	1 × 10^−176^
	Cytochrome bc1	AJHOBMPL_08379	−1.78	5.09 × 10^−23^
	Cytochrome c oxidase	AJHOBMPL_02024	−1.75	5.33 × 10^−14^
	ATP synthase	AJHOBMPL_06406	−3.42	1.55 × 10^−50^

### The pathway of fatty acid biosynthesis and DNR biosynthesis

In the present study, the genes involved in fatty acid biosynthesis were significantly upregulated in the DM-F group, as shown in Table 3. However, this extensive upregulation of fatty acid biosynthesis may be detrimental to the biosynthesis of DNR as the initial materials required for DNR biosynthesis may become depleted. Sequencing results revealed that the genes involved in DNR biosynthesis were completely repressed when DM was cultured in F media. This suggests that the reducing power and energy derived from glycolysis and the TCA cycle may be preferentially utilized for fatty acid biosynthesis and glucuronidation processes under carbon catabolite repression (CCR) conditions.

**Table 3:** Changes in transcript expression of genes related to fatty acid biosynthesis and DNR biosynthesis.

Pathway	Definition	ID	Log2Fold change	Adjusted-*p*-value
Fatty acid biosynthesis	Acetyl-CoA carboxylase, AccD	AJHOBMPL_08492	1.88	6.99 × 10^−6^
	Malonyl CoA-acyl carrier protein transacylase, FabD	AJHOBMPL_02862	2.87	6.75 × 10^−28^
	3-oxoacyl-[acyl-carrier-protein] synthase, FabH	AJHOBMPL_02863	3.03	2.63 × 10^−35^
	Acyl carrier protein, AcpP	AJHOBMPL_02864	2.69	7.34 × 10^−24^
	3-oxoacyl-[acyl-carrier-protein] synthase FabF	AJHOBMPL_02865	2.80	1.23 × 10^−33^
DNR biosynthesis	Transcriptional regulatory DnrO	AJHOBMPL_06356	−1.93	2.01 × 10^−28^
	Transcriptional regulatory DnrI	AJHOBMPL_06344	−2.33	1.68 × 10^−30^
	Rhodosaminyltransferase DnrS	AJHOBMPL_06323	−4.01	1.46 × 10^−72^
	Ketoacyl synthase DpsA	AJHOBMPL_06332	−4.54	3.3 × 10^−99^
	Ketoacyl reductase DpsE	AJHOBMPL_06334	−4.18	1.28 × 10^−95^
	Cytochrome monooxygenase DoxA	AJHOBMPL_06343	−3.40	4.3 × 10^−107^

**Figure 5:**
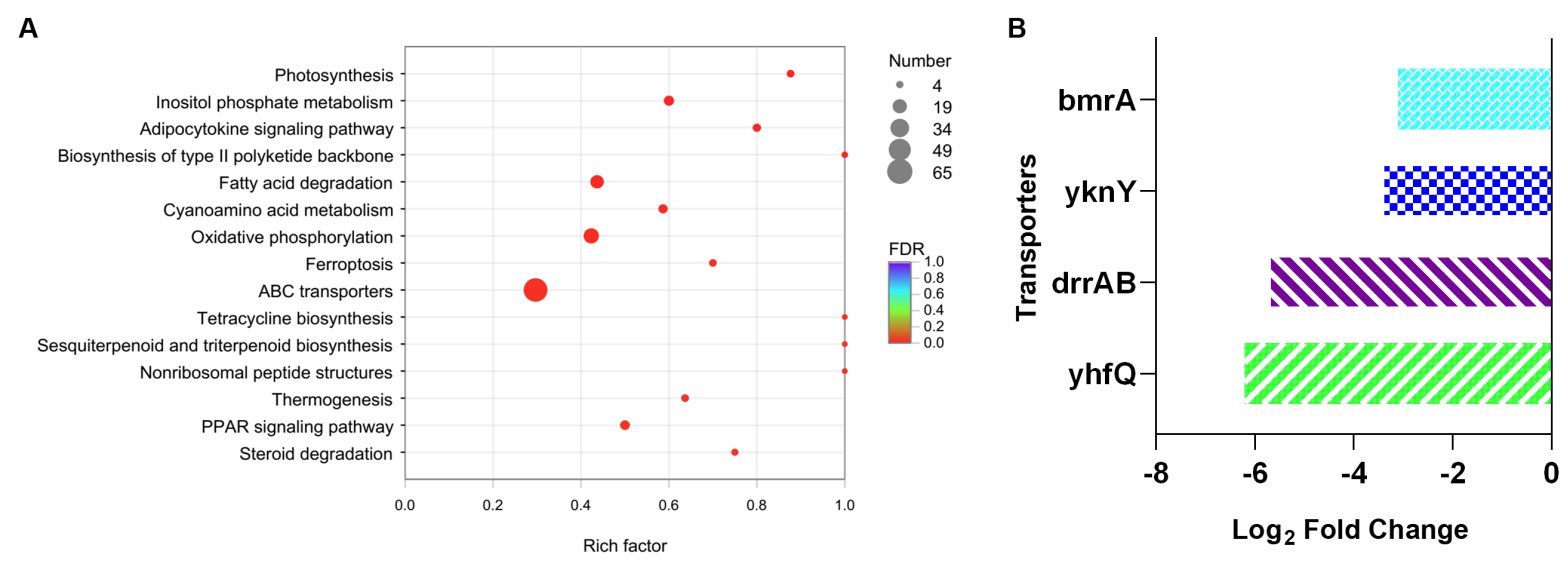
Analysis of transcript expressed genes associated with ABC transporter. **(A)** KEGG enrichment analysis of downregulated genes of DM-F vs. DM-Y. **(B)** Log_2_FoldChange values of genes involved in ABC transporter.

Furthermore, KEGG analysis was applied to investigate the variation of metabolic process between DM-F group and DM-Y group. The functional genes were annotated to different metabolic process, and totally 65 genes were involved in the ABC transporters metabolic pathway, which was significantly downregulated in the F group (Figure 5A). This includes the repression of genes encoding efflux pumps such as DNR export transporters DrrAB, as well as the bacterial multidrug efflux pump YknY, YhfQ and BmrA (Wiseman and Jault, 2016) (Figure 5B). The significant downregulation of efflux transporter genes in F media might also promote the intracellular biotransformation of anthraquinones into glucuronidated products.

## Discussion

Natural glucuronides are widely distributed in plants, where glucuronic acid is attached to aglycons derived from plants. These glucuronides, such as baicalein-7-O-β-glucuronide, quercetin-3-O-β-glucuronide, and epicatechin glucuronide, are known for their health-promoting activities. Glycyrrhizin, which contains two glucuronic acid moieties, is widely utilized as an anti-hepatitis agent and sweetener (Yonekura-Sakakibara and Hanada, 2011). Additionally, glucuronidated flavonoids have been reported to exhibit stronger inhibitory activity against amyloid β compared to their aglycon counterparts (Ben Hmidene et al., 2017). These natural glucuronides highlight the importance of glucuronidation in plant metabolism and their potential therapeutic applications.

Nowadays, most of the glucuronosyltransferases (UGTs) responsible for glucuronation are derived from mammalian cell systems or plants, while the microbial glucuronation process remains relatively understudied. Microbial glucuronation has typically been associated with the biosynthesis of saccharides, with limited biocatalyst activity. However, recent research has revealed that *S. chromofuscus* ATCC 49982 has the ability to convert various plant polyphenols into different glucuronides (Ren et al., 2022). *Streptomyces* is well-known for its capacity to biosynthesize secondary metabolites. In our study, we unexpectedly discovered that *S. coeruleorubidus* DM, which is primarily recognized as an industrial producer of DNR, exhibits highly efficient anthraquinone glucuronidation ability under carbon catabolite repression conditions. This finding opens up new possibilities for exploring microbial glucuronidation processes.

Carbon catabolite repression (CCR) is closely linked to secondary metabolite production in *Streptomyces*. Glucose, in particular, has been found to have a negative impact on antibiotic production. When high concentrations of glucose and other carbohydrates are added to *Streptomyces* culture media, it often leads to downregulation or even complete inhibition of antibiotic production (Romero-Rodriguez et al., 2016b). For instance, in *S. coelicolor*, high levels of glucose inhibit the production of actinorhodin (ACT) and undecylprodigiosin (RED) by limiting the availability of precursors (Ruiz et al., 2010). Anthracycline production is negatively regulated by excessive glucose in *S. peucetius* (Guzman et al., 2005). In our study, DNR was observed to completely abolished in *S. coeruleorubidus* DM in F media, which could be attributed to the complete repression of DNR biosynthesis by the high concentration of carbohydrates present in F media. The effect of different glucose concentration on DNR production was also preliminarily investigated, the results showed that when the glucose concentration reached 6 g/L (data not shown), the production of DNR appeared to be completely suppressed. It is evident that the production of anthracyclines is highly sensitive to the concentration of carbohydrates.

DM was observed to have high glucuronidation efficacy on emodin, alizarin, purpurin and anthraflavic acid, however no glucuronides of aloe-emodin was produced. The transformation results indicated that DM specifically recognizes the hydroxyl group on the anthraquinone skeletons. Additionally, no glucuronides of 1,8-dihydroxyanthraquinone were observed, possibly due to hydrogen bonding between the carbonyl group at position 9 and the hydroxyl group at carbon-1 or carbon-5 (Fang et al., 2019). Furthermore, neither 2-aminoanthraquinone nor 1,4-diaminoanthraquinone were observed to be converted into their corresponding glucuronides. This suggests that DM appears to catalyze only anthraquinone O-glucuronidation.

**Figure 6:**
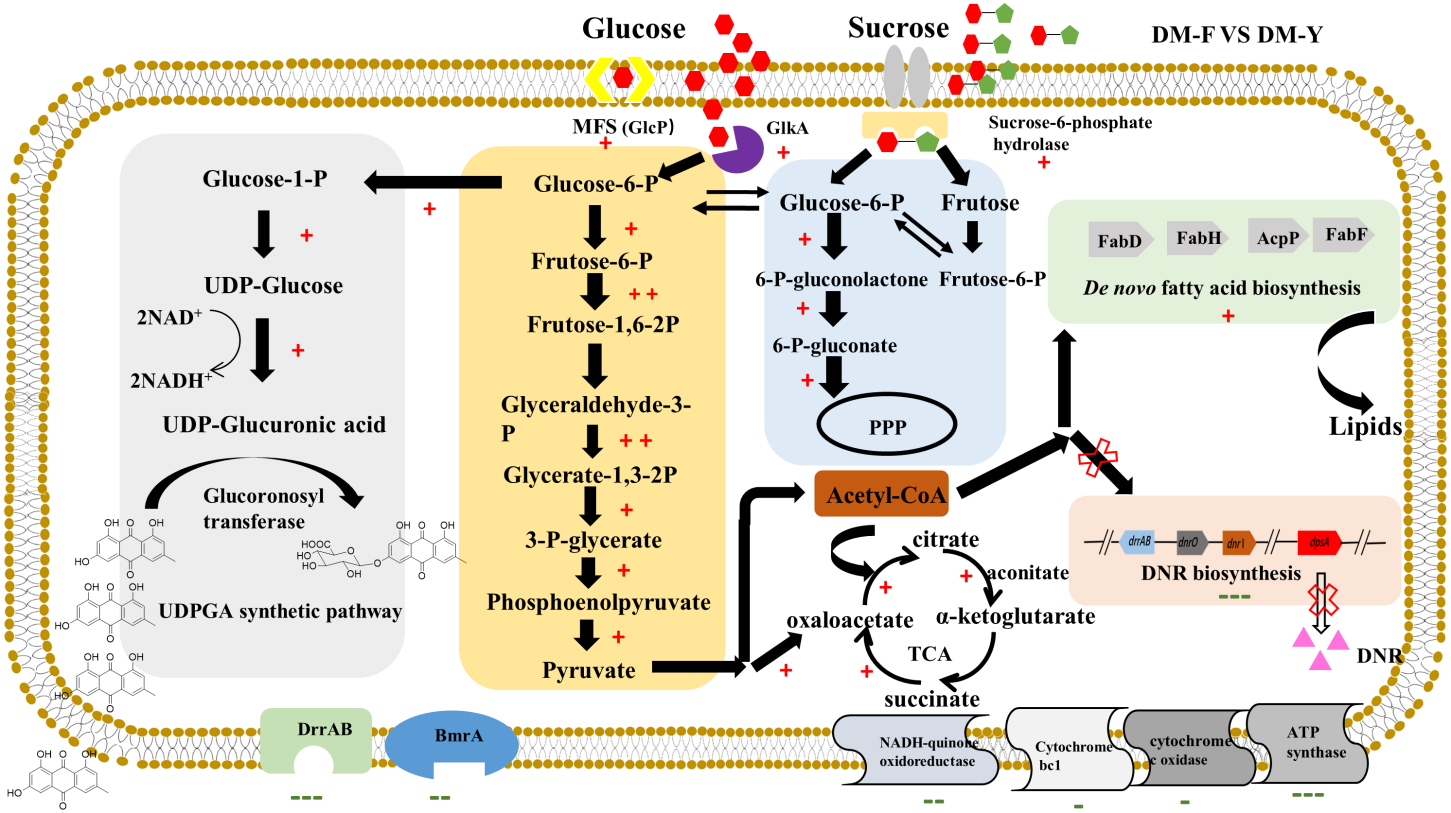
Probable metabolic mechanism of DM under CCR condition for efficient glucuronidation through comparative transcriptome analysis. +Indicates gene expression of log_2_FC > 1, ++indicates gene expression of log_2_FC > 3, −indicates gene expression of log_2_FC < −1, −−indicates gene expression of log_2_FC < −3, −−−indicates gene expression of log_2_FC < −5.

Interestingly, the glucuronidation process in our study appears to be driven by carbon catabolite repression (CCR). The putative metabolic mechanism underlying efficient glucuronidation under CCR conditions was analyzed by comparative transcriptomic analysis in Figure 6. Our results showed that the genes involved in the glucuronidation process were mostly upregulated, indicating their active involvement in the biotransformation function. Furthermore, our results also demonstrated that the biosynthetic pathway of fatty acids was significantly increased under CCR conditions. In many *Streptomyces* species, *de novo* synthesized fatty acids serve a dual role in constructing membrane phospholipids and acting as precursors for polyketide compound biosynthesis (Gago et al., 2011). The biosynthesis of fatty acids begins with the carboxylation of acetyl-CoA by acetyl-CoA carboxylase (AccD), resulting in the formation of malonyl-CoA, which serves as the extender unit. These metabolites are crucial raw materials for DNR production. However, under CCR, these resources are diverted towards fatty acid biosynthesis, leading to their depletion for DNR production. Emodin and other anthraquinones are lipophilic compounds, allowing them to cross the lipid bilayer of the cell membrane and enter the cytoplasm. We observed a significant downregulation of efflux transporter genes, which could enhance the intracellular concentration of the substrate and promote the biotransformation process. The permeability barrier of the cell envelope also plays a crucial role in the biotransformation activity. Most genetic engineering hosts, such as *E. coli*, have an outer membrane structure that may restrict the passage of substrates (Ni and Chen, 2004). In contrast, our study utilizes *Streptomyces coeruleorubidus* DM, a Gram-positive bacterium that lacks an outer membrane. This characteristic of *Streptomyces* may contribute to the high-efficiency anthraquinone glucuronidation process observed in our study. These findings highlight the importance of cellular permeability and efflux transporter regulation in the biotransformation of anthraquinones, and suggest that the unique characteristics of *Streptomyces* species can be advantageous for efficient glucuronidation processes. Overall, this study represents the first successful utilization of carbon catabolite repression for natural product biotransformation in *Streptomyces*. The findings open up new possibilities for the economical and environmentally-friendly production of bioactive compounds through bio-catalytic approaches.

## Data availability statement

The raw transcriptome sequence data has been deposited in the NCBI database under accession number PRJNA755635.

## Author contributions

CT: Writing – original draft, Methodology, Investigation. QW: Writing – original draft. JJ: Writing – original draft, Methodology. ZZ: Writing – original draft, Methodology. BY: Writing – original draft, Methodology. RZ: Writing – original draft, Formal analysis. SJ: Writing – review & editing, Supervision. TY: Writing – review & editing, Writing – original draft, Conceptualization.

## Footnotes

^1^ www.majorbio.com
