# Supplementary material for: Utilization of carbon catabolite repression for efficiently biotransformation of anthraquinone O-glucuronides by Streptomyces coeruleorubidus DM
Source: Front Microbiol. 2024 Apr 16;15:1393073. doi: 10.3389/fmicb.2024.1393073 (PMC11058961; doi:10.3389/fmicb.2024.1393073)
Supplement: Supplementary file 1 [file Table_1.DOCX]

**Utilization of Carbon Catabolite Repression for Efficiently Biotransformation of**

**Anthraquinone O‑Glucuronides by *Streptomyces coeruleorubidus* DM**

Chen Tao^1^, Quyi Wang^1^, Junyang Ji^1^, ZiYue Zhou^1^, Bingjie Yue^1^, Ran Zhang^1^, Shu Jiang^1,2^, Tianjie Yuan^1^*

^1^ School of Pharmacy, Nanjing University of Chinese medicine, Nanjing, 210023, China

^2^ Jiangsu collaborative innovation center of Chinese medical resources industrialization, Nanjing University of Chinese medicine, Nanjing, 210023, China

*Correspodning author: Tianjie Yuan

*E-mail address*: [YTJ@njucm.edu.cn](mailto:YTJ@njucm.edu.cn)

**Fig. S1 ^1^ H, ^13^C of NMR of purpurin-*O*- glucuronide**

**Fig.S2 ^1^ H, ^13^C of NMR of alizarin-*O*- glucuronide**

**Fig. S3 ^1^ H, ^13^C of NMR of anthraflavic acid-*O*- glucuronide**

**Fig. S4 ^1^ H NMR of emodin-*O*- glucuronide**
